# Impact of the *Supporting Physical Activity in the Childcare Environment* (SPACE) intervention on preschoolers’ physical activity levels and sedentary time: a single-blind cluster randomized controlled trial

**DOI:** 10.1186/s12966-017-0579-7

**Published:** 2017-09-07

**Authors:** Patricia Tucker, Leigh M. Vanderloo, Andrew M. Johnson, Shauna M. Burke, Jennifer D. Irwin, Anca Gaston, Molly Driediger, Brian W. Timmons

**Affiliations:** 1 School of Occupational Therapy, Faculty of Health Sciences, University of Western Ontario, 1201 Western Road, Elborn College, Room 2547, London, ON N6G 1H1 Canada; 2 Health and Rehabilitation Sciences, Faculty of Health Sciences, University of Western Ontario, London, ON Canada; 3 School of Health Studies, Faculty of Health Sciences, University of Western Ontario, London, ON Canada; 4 School of Kinesiology, Faculty of Health Sciences, University of Western Ontario, London, ON Canada; 5 Child Health & Exercise Medicine Program, McMaster University, Hamilton, Canada

**Keywords:** Physical activity, Sedentary time, Childcare, Preschooler, Outdoor playtime, Early years, Intervention

## Abstract

**Background:**

Physical activity levels among preschoolers in childcare are low and sedentary time high. The *Supporting Physical Activity in the Childcare Environment* (SPACE) intervention had three components: 1. portable play equipment; 2. staff training; and, 3. modified outdoor playtime (i.e., shorter, more frequent periods). This study aimed to examine the effectiveness of the SPACE intervention on preschoolers’ physical activity levels and sedentary time during childcare hours (compared to standard care).

**Methods:**

Via a single-blind cluster randomized controlled trial, 338 preschoolers (39.86 ± 7.33 months; 52% boys) from 22 centre-based childcare facilities (11 experimental, 11 control) were enrolled. Preschoolers wore an Actical™ accelerometer for 5 days during childcare hours at baseline, post-intervention, and 6- and 12-month follow-up, and were included in the analyses if they had a minimum of two valid days (5 h each day) at baseline and one additional time point. Intervention effectiveness was tested using a linear mixed effects model for each of the four outcome variables (i.e., sedentary time, light physical activity [LPA], moderate-to-vigorous physical activity [MVPA], and total physical activity [TPA]). Fixed effects were further evaluated with *t*-tests, for which degrees of freedom were estimated using a Satterthwaite approximation.

**Results:**

One hundred and ninety-five preschoolers were retained for analyses. The intervention did not significantly impact LPA. MVPA was significantly greater among children in the experimental group when comparing post-intervention to pre-intervention, *t*(318) = 3.50, *p* = .0005, but no intervention effects were evident at 6- or 12-month follow-up. TPA was significantly greater for children in the intervention group at post-intervention when compared to pre-intervention, *t*(321) = 2.70, *p* = .007, with no intervention effects evident at later time periods. Finally, sedentary time was significantly lower among preschoolers in the experimental group when comparing post-intervention to pre-intervention, *t*(322) = 2.63, *p* = .009, with no significant effects at follow-up.

**Conclusions:**

The SPACE intervention was effective at increasing MVPA and TPA among preschoolers, while simultaneously decreasing sedentary time. The ability of the SPACE intervention to target higher intensity activity is promising, as MVPA levels have been documented to be low in centre-based childcare. The changes in physical activity were not sustained long term (6- or 12-month follow-up).

**Trial registration:**

ISRCTN70604107 (October 8, 2014).

## Background

Physical activity participation among preschoolers (defined herein as 2.5–5 years) in centre-based childcare facilities has been consistently reported as low [1–4]; likewise, the prevalence of sedentary time has been noted as high in this environment [5–7]. In fact, previous research contends that preschoolers in childcare centres spend only 1.5 min/h in moderate-to-vigorous physical activity (MVPA), devoting the majority of their time (40.6 min/h) to sedentary pursuits [1]. Unfortunately, these trends could have potentially devastating impacts on the health and development of young children. Physical activity has been identified as positively impacting cardiovascular health, and is associated with improved weight status and better psychosocial and cognitive development [8, 9]. Similarly, excess sedentary time has been linked with a risk for increased adiposity and poorer psychosocial health and cognitive development [10, 11]. Canadian Physical Activity Guidelines recommend the accumulation of 180 min of physical activity (at any intensity) per day among this young population [12], moving towards 60 min of MVPA by the age of 5 years. Canada’s Sedentary Behaviour guidelines (for children aged 2–4 years), encourage minimizing sedentary pursuits for overall healthy growth and development, and specifically advise against more than one hour of daily screen time [13]. As such, early intervention is needed to support the development of appropriate physical activity and sedentary levels among preschoolers.

Typified by providing care to young children in a classroom-like setting, centre-based childcare has received notable attention in the literature, particularly regarding obesity-related behaviours (such as physical activity and screen-viewing) [14, 15]. Often recognized as an obesogenic and sedentary environment [2, 6, 16], researchers suggests that the childcare environment accounts for roughly 50% of the variation in preschoolers’ physical activity [3, 17]. More specifically, a number of childcare factors, including portable play equipment and supportive staff behaviours have been recognized as strong correlates to physical activity participation [2]. The majority of Canadian preschoolers attend some form of non-parental care [18, 19]. This fact, combined with previous findings that those who are cared for in this particular environment (compared to those cared for by a parent or relative) are at a higher risk for becoming obese in later childhood [20], make centre-based childcare facilities an appropriate, if not necessary, venue for targeting health promotion efforts.

Evidence-informed action is needed to improve and support the physical activity levels and reduce the sedentary time of preschoolers in childcare. Recently, steps have been taken to understand the effective qualities of physical activity interventions targeting this population. Via systematic review and meta-analysis, Ward et al. [21] and Gordon et al. [22], both highlighted the effectiveness of intervening in childcare centres and offering environmental modifications (e.g., portable play equipment, floor markings) as successful approaches to improving young children’s physical activity levels. Moreover, the importance of outdoor play was also supported [22]. This is no surprise given that, generally, young children exhibit higher levels of physical activity when outdoors [23–27]. This sentiment is echoed by the 2015 ParticipACTION Position Statement on Outdoor Active Play which highlighted the physiological and psychosocial benefits gained by children playing outside [28]. Specific to the childcare environment, a recent study by Vanderloo et al. [27] reported that preschoolers accumulated significantly higher rates of physical activity and significantly lower sedentary time during outdoor play periods than when indoors at childcare. Moreover, preschoolers’ activity levels have been shown to be highest when they are first exposed to their outdoor environment (i.e., within the first 10 min), with activity levels declining with increased duration of outdoor play [29, 30]. Given the positive impact of outdoor playtime on this population’s activity levels, interventions adopting this approach show great promise.

At an international level, several interventions have recently been undertaken in the childcare setting [31–40]. These studies underscore not only the appropriateness of the childcare venue for intervention, but also the pressing need to improve activity behaviours of young children enrolled in these settings. With consolidated evidence now available regarding effective strategies for improving preschoolers’ physical activity levels [21, 22], and with consideration of childcare providers’ perspectives regarding the barriers and facilitators to engaging preschoolers in physical activity in childcare [41, 42], the 8-week *Supporting Physical Activity in the Childcare Environment* (SPACE) intervention was created. The SPACE intervention was developed primarily to support preschoolers’ physical activity, and secondarily to decrease sedentary time, in centre-based childcare via a unique combination of environmental modifications, staff training, and modified outdoor playtime periods.

The purpose of this randomized controlled trial (RCT) was to examine the effectiveness of the SPACE intervention with regard to increasing preschoolers’ physical activity levels and decreasing sedentary time during childcare hours. It was hypothesized that preschoolers assigned to the experimental condition would display higher rates of physical activity and lower rates of sedentary time compared to preschoolers in the control condition, from pre- to post-intervention, and at 6- and 12-month follow-up periods.

## Methods

### Description of SPACE intervention

The SPACE study was an evidence-informed physical activity intervention comprised of the following components: 1. environmental modifications (i.e., provision of novel portable play equipment including balls, hula hoops, a hop-scotch mat, obstacle course, stepping domes, ribbon wands, and hop-along bouncers); 2. staff training (i.e., one 4-h physical activity training session for staff and directors that emphasized the importance of shorter bouts of physical activity and reduced sedentary time, recommendations for overcoming obstacles, explanation of the Canadian physical activity and sedentary behaviour guidelines, and provided examples of activities that could be implemented in childcare); and, 3. modified outdoor playtime (i.e., re-structuring the provincially required two 60-min outdoor sessions into four 30-min periods). Given recent evidence to suggest that preschoolers’ activity levels are highest during their first 10 min outdoors, and consequently, that simply extending outdoor playtime may not be adequate for promoting physical activity [29], the SPACE intervention consisted of a modified outdoor playtime schedule to increase the frequency, but not the duration, of unstructured outdoor playtime provided to young children in childcare. See Tucker et al. [43] for additional details regarding each component of the intervention and supporting evidence.

### Study design and recruitment

**Fig. 1:**
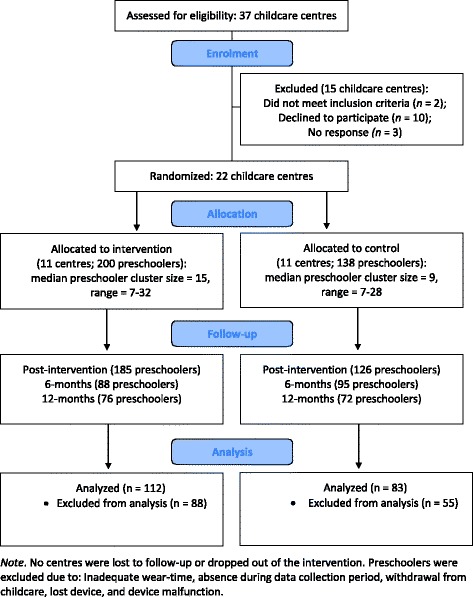
*CONSORT flow diagram for the SPACE intervention*

A detailed methodological account has been published elsewhere [43]. Informed by the PRECEDE-PROCEED model for health promotion program planning [44], and methodologically designed and implemented in accordance with the Consolidated Standards of Reporting Trials (CONSORT) statement [45], a single-blind cluster RCT was conducted. Childcare centres were eligible if they had at least one preschool classroom, if the staff and children were English-speaking, and if the centre director and childcare staff of the eligible classrooms were willing to participate. All recruitment and randomization activities were conducted by the project coordinator. Twenty-two centre-based childcare facilities in London, Ontario, Canada were randomly selected and agreed to participate (59%; see Fig. 1 for participation rates). The director of each childcare facility was contacted via phone; once verbal consent was received from all 22 centres, each centre was randomly assigned to either the experimental or control condition (using a blocked design). Centre start dates were staggered over four months during spring and summer, making it logistically impossible to perform baseline measures prior to randomization. No centres withdrew from the study at any time. Only centres assigned to the experimental condition had childcare staff deliver the SPACE intervention (i.e., providing four 30-min daily outdoor periods and offering the supplied portable play equipment), while centres enrolled in the control condition continued their typical daily curriculum and programming, including regular outdoor playtime periods (i.e., two 60-min periods). This study and all related documents received ethical approval from the University of Western Ontario’s Research Ethics Board (REB# 105779) and was assigned an International Standard Randomized Controlled Trial Number (ISRCTN 70604107).

### Participants

Parents/guardians of typically developing children (i.e., free of any chronic disease or developmental issue) within the preschool classroom(s) of the selected childcare centres were invited to participate. Preschoolers were eligible if they were between the ages of 2.5 and 4 years and had a parent/guardian who spoke and understood English to provide informed consent. Based on the power calculation conducted for the SPACE study [43], the recruitment goal was 348 preschoolers from 22 childcare centres.

### Data collection

**Fig. 2:**
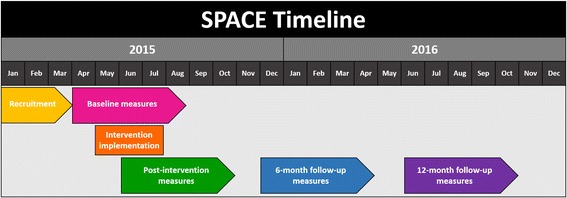
The *Supporting Physical Activity in the Childcare Environment* (SPACE) Intervention Timeline

Data collection occurred between March 2015 and October 2016, with the intervention being implemented, in a staggered format, from April to July 2015 (see Fig. 2 for SPACE timeline). Measurements were completed for preschoolers in both the experimental and control condition at baseline (i.e., week 0), immediately post-intervention (i.e., week 8), and at 6- and 12-month follow-up. Trained in conducting anthropometric measurements (inter-rater reliability, *r* = .99), research assistants (*n* = 2), who were blind to group assignment, completed all measurements. Research staff visited participating childcare centres prior to the onset of each data collection period to distribute the accelerometers (including instruction sheets and daily wear-time logs) and questionnaire packages.

### Tools

Accepted as a valid and reliable tool for assessing young children’s activity behaviours [46, 47], Actical™ accelerometers (Z and B series; Phillips Respironics, Bend, Oregon) were used to objectively measure participants’ physical activity and sedentary time. Worn on participants’ right hip and secured with an elastic waist band, preschoolers were asked to wear the accelerometer for five consecutive days, during childcare hours only, at each of the four time points. Childcare staff were instructed to place the devices on the children’s right hip when they arrived at the centre and to remove them at end-of-day prior to leaving for home. Staff were also asked to keep a daily wear-time log for each participating child.

Accelerometers were programmed using a 15-s epoch length, and in line with the Canadian Health Measures Survey [48], cut-points (all divided by four to match the time sampling interval used in the present study) by Adolph and colleagues [49] were used: light physical activity, LPA: ≥25 ≤ 287.25 counts⋅15 s^−1^⋅epoch^−1^; MVPA ≥287.5 counts⋅15 s^−1^⋅epoch^−1^; total physical activity, TPA: ≥25 counts⋅15 s^−1^⋅epoch^−1^. Sedentary cut-points [50] were also applied to the collected accelerometer data: ≤24.75 counts⋅15 s^−1^. Rates (min/h) were calculated to account for differences across groups (i.e., varying wear-times due to differences in childcare hours and attendance).

Prior to each data collection time-point, anthropometric measurements were taken at the childcare centre to calculate preschoolers’ body mass index (BMI) percentiles. Height was measured to the nearest 0.1 cm using a Seca 214 “Road Rod” Portable Stadiometer, weight was measured to the nearest 0.1 kg using a Tanita 700-TBF300GS Body Fat Analyzer w/Goal Setter scale, and waist circumference was measured to the nearest 0.1 cm at the child’s naval using an anthropometric measuring tape. Prior to these measurements, participants were asked to remove their shoes and any heavy clothing.

A demographic questionnaire was also administered to the parents/guardians of participating children. This questionnaire was completed at baseline only and was used to collect information on potential correlates of participants’ activity levels. Such items included: age, sex, and ethnicity of preschooler; yearly family income; parent/guardian education level; family status, and child’s participation in extra-curricular activities outside of childcare hours.

### Data analysis

To compare demographic variables between the experimental and control groups, continuous variables were evaluated using independent sample *t*-tests, while categorical variables were explored using Pearson chi-square calculations.

Accelerometer data were downloaded using device specific software (Actical™ version 3.10) and analyzed at an epoch length of 15 s. Files were then uploaded to *KineSoft* (version 3.3.62; Loughborough, United Kingdom) to assess the quality of the data files for each participant at each time point. Only participants with a minimum of 2 valid days (where 5 h of wear-time equated to a valid day) at baseline and one additional time point, were retained for analyses. Non-wear time was defined as 20 min of consecutive zeros [51, 52]. See Table 1 for additional details regarding accelerometer data collection and compliance.

**Table 1:** Accelerometer compliance for the SPACE Study

	Pre-Intervention	Post-Intervention	6-Month Follow-Up	12-Month Follow-Up
Number of Devices Distributed	335	313	180	141
Number of Devices Not Returned	1	-	-	1
Number of Devices that Malfunctioned	4	-	2	-

LPA, MVPA, TPA, and sedentary time were the outcomes of interest for the present study. Each outcome variable was evaluated within a linear mixed effects model, with group (experimental versus control) and time (baseline, post-intervention, 6-month follow-up, and 12-month follow-up) as fixed effects. This analytic approach reduces concerns regarding missing data, as participant error is modeled within the design as a random effect. This approach also allows for the testing of cluster effects (i.e., the childcare centre from which the individual was recruited), to ensure that the randomization method did not have a systematic effect on the outcome. To identify the model of best fit, the following models were tested: 1. a “null model”; 2. a “main effects only” model (i.e., a model in which group and time were not allowed to correlate); 3. an “interaction” model (i.e., a model in which group and time were allowed to correlate); and, 4. a model that considered cluster effects. Models 1 through 3 were tested hierarchically (i.e., Model 2 was compared with Model 1, and Model 3 was compared with Model 2). Model 4 (the model that included cluster effects) was, for each dependent variable, compared against the best fitting model that did not include cluster effects. In each case, the model comparison was conducted using a Pearson chi-square test. With the reporting of the model of best fit, parameters were compared to the reference group using a *t*-test, and degrees of freedom were estimated with a Satterthwaite approximation [53]. All models were fitted using the lme4 package [54] in R [55], and the *t*-tests used in the interpretation of model effects were computed using the ImerTest package [56].

## Results

### Description of sample

A total of 338 preschoolers (39.86 ± 7.33 months; 52% boys) enrolled in the SPACE study (response rate of 73%). Children in the treatment group (*M* = 40.61, *SD* = 7.31) were significantly older than children in the control group (*M* = 38.72, *SD* = 7.24), *t*(307) = 2.23, *p* = 0.026. There was no significant difference in BMI percentile between the treatment group (*M* = 58.21, *SD* = 27.78) and the control group (*M* = 56.03, *SD* = 29.69), *t*(247) = .59, *p* = 0.56. When our accelerometer wear time parameters were applied (2 valid days), 195 participants were retained for analyses. Age (months) differed significantly between compliant (*M* = 40.53; *SD* = 7.76) and non-compliant (*M* = 38.93; *SD* = 6.6) participants *t*(298) = 1.95, *p* = 0.052; however, no other significant differences in baseline characteristics were found between children with incomplete or complete baseline PA data in either group. See Table 2 for full demographic information of the participating preschoolers.

**Table 2:** Descriptive characteristics of enrolled preschoolers

Variable	Control	Intervention	*p*-value
Age (months), *M* (*SD*)	38.72 (7.24)	40.61 (7.31)	.03
Sex (male/female), *n*	76/62	102/98	.46
BMI Percentiles, *M* (*SD*)	56.03 (29.69)	58.21 (27.78)	.26
Hours in Childcare (hours)			.82
< 10	3	4	
10–19	8	18	
20–29	16	23	
30+	95	142	
Ethnicity			.48
Caucasian	87	142	
African Canadian	1	4	
Aboriginal	5	4	
Arab	2	3	
Latin-American	3	6	
Asian	7	7	
Other	19	19	
Family Income			.65
< $20,000	11	9	
$20,000–$39,999	13	14	
$40,000–$59,999	10	14	
$60,000–$79,999	12	11	
$80,000–$99,999	8	16	
$100,000–$119,999	10	15	
$120,000–$149,999	11	20	
> $150,000	28	45	
Highest Level of Education			.92
Elementary	1	2	
Secondary	9	9	
College	42	62	
University	42	60	
Graduate School	28	47	
Family Situation			.45
Single Parent	22	23	
Double Parent	94	153	
Other	2	2	

Frequencies (*n*) unless otherwise noted. Groups were compared using independent *t*-tests for continuous data and *x* ^*2*^ tests were used for categorical

### Effects of the SPACE intervention on physical activity and sedentary time

**Fig. 3:**
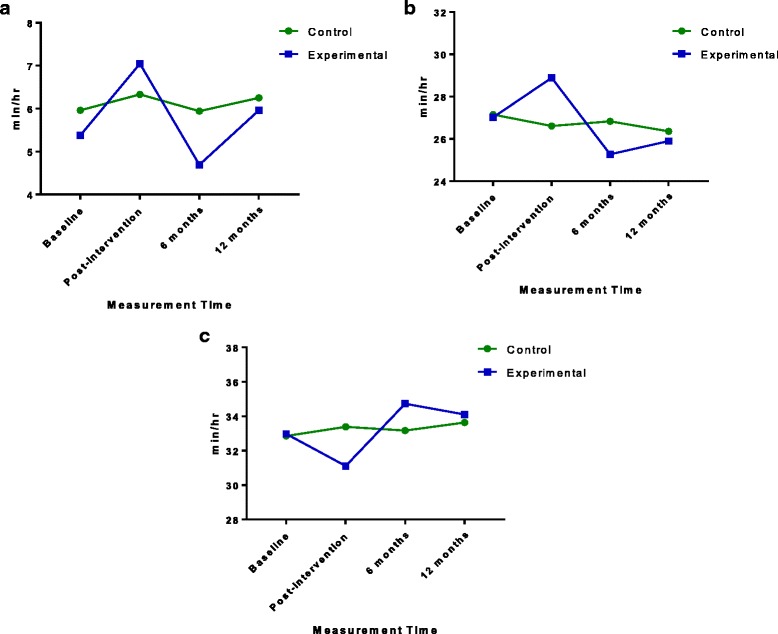
**a**, **b**, **c** Preschoolers’ moderate-to-vigorous physical activity (**a**), total physical activity (**b**), and sedentary time (**c**) in minutes per hour across four measurement times for experimental and control groups

Means and standard deviations for physical activity intensities and sedentary time, separated by time and group, are presented in Table 3. Rates of MVPA, TPA, and sedentary time are displayed in Fig. 3. The results of the linear mixed-effects model-testing is presented in Table 4. Cluster effects were non-significant for all four outcome variables.

**Table 3:** Means (Standard Deviations) of physical activity intensities (min/hr) for preschoolers in the control and experimental conditions

Time	LPA		MVPA		TPA		Sedentary Time	
	Control	Exp.	Control	Exp.	Control	Exp.	Control	Exp.
Baseline	21.20 (3.69)	21.64 (2.93)	5.96 (2.32)	5.38 (2.12)	27.15 (5.13)	27.02 (4.15)	32.85 (5.25)	32.98 (4.15)
Post	20.28 (3.23)	21.85 (3.07)	6.33 (2.63)	7.05 (2.63)	26.61 (4.86)	28.89 (4.38)	33.39 (4.86)	31.11 (4.38)
*Diff (95% CI)*	0.91 (−0.29 to 2.11)		1.28 (0.57 to 1.99)		2.15 (0.58 to 3.72)		−2.13 (−3.72 to −0.54)	
6 month	20.89 (3.88)	20.58 (5.04)	5.94 (2.11)	4.69 (1.86)	26.83 (4.80)	25.27 (6.37)	33.17 (4.80)	34.73 (6.37)
*Diff (95% CI)*	−0.79 (−2.30 to 0.72)		−0.58 (−1.48 to 0.32)		−1.38 (−3.36 to 0.60)		1.40 (−0.60 to 3.40)	
12 month	20.11 (2.74)	19.93 (3.73)	6.25 (2.16)	5.96 (2.57)	26.36 (3.68)	25.90 (5.63)	33.64 (3.68)	34.10 (5.63)
*Diff (95% CI)*	−0.87 (−2.54 to 0.80)		−0.19 (0.51) (−1.19 to 0.81)		−1.04 (−3.22 to 1.14)		1.16 (−1.05 to 3.37)	

*LPA* light physical activity, *MVPA* moderate-to-vigorous physical activity, *TPA* total physical activity, *Exp*. experimental condition

Reported differences for each time period represent the difference between groups (experimental minus control), when compared with baseline

**Table 4:** The Effect of the SPACE Intervention on LPA, MVPA, TPA, and Sedentary Time

Model	LPA		MVPA		TPA		Sedentary Time	
^a^Main effects	χ^2^(4) = 13.24	*p* = .01	χ^2^(4) = 43.67	*p* < .0001	χ^2^(4) = 14.32	*p* = .006	χ^2^(4) = 13.72	*p* = .008
^b^Interaction	χ^2^(3) = 6.52	*p* = .09	χ^2^(3) = 19.44	*p* = .0002	χ^2^(3) = 14.92	*p* = .002	χ^2^(3) = 14.70	*p* = .002

*LPA* light physical activity, *MVPA* moderate-to-vigorous physical activity, *TPA* total physical activity

^a^tested against the null model

^b^tested against the main effects model

The results presented in Table 4 suggest that the intervention was effective at improving preschoolers’ MVPA and TPA, and decreasing sedentary time, indicated by a statistically significant interaction between group (i.e., experimental vs. control) and time (i.e., baseline and post-intervention only). For all three of these variables, the experimental group displayed a significant difference between baseline and post-intervention as compared to the control group, but no significant effects were found at 6- or 12-month follow-up. Both MVPA, *t*(317) = 3.47 (*p* = 0.0005), and TPA, *t*(321) = 2.70 (*p* = 0.007), were significantly higher post-intervention, while sedentary time was significantly less post-intervention, *t*(322) = 2.632 (*p* = 0.009).

## Discussion

The purpose of this study was to examine the impact of the SPACE intervention on the physical activity levels and sedentary time of preschoolers enrolled in centre-based childcare. The SPACE study is unique because it entails a combination of evidence-informed components known to be associated with preschoolers’ physical activity and sedentary time while in childcare. The results of this study suggest that the 8-week SPACE intervention was effective at increasing preschoolers’ MVPA and TPA levels and at decreasing sedentary time from pre- to post-intervention. However, changes to activity behaviours and sedentary time were not sustained (at 6- or 12-month follow-up) when the intervention was removed.

A number of physical activity interventions have transpired in childcare centres with varying success. Specifically, Bellows et al. [57], who implemented the Mighty Moves® intervention (consisting of 15–20 min classroom lessons focused on gross motor skill acquisition for 18 weeks) found no significant effect of their intervention on preschoolers’ physical activity. Similarly, Jones and colleagues [58] implemented the Jump Start program which also focused on preschoolers’ gross motor skills and comprised professional development for childcare staff in addition to structured and unstructured activities for children. While the results of their intervention were not significant, improvements in activity behaviours were reported for children enrolled in the intervention. In a large Belgian population, De Creamer and colleagues found the ToyBox intervention, which transpired in Kindergarten classrooms, but included parents and families in the intervention, had a significant effect on preschoolers’ activity, but noted this effect was strongest for boys and for preschoolers from higher socioeconomic classes [59]. Finally, Goldfield et al. [34] adopted a train-the-trainer approach and coupled it with a resource manual which provided childcare staff with guidance on facilitating structured and unstructured physical activities. Preschoolers in the intervention group displayed greater increases in minutes of TPA and LPA, but not MVPA at 6-months [34]. Despite the inconsistent findings reported in these 4 studies, childcare centres still maintain promise as an appropriate venue for intervening given the substantial portion of their day that young children spend in these venues [19]. Moreover, young children have been noted to accumulate more MVPA in care, compared to at home [60], which supports the feasibility of higher intensity physical activity in the childcare environment.

The SPACE intervention involved two common intervention components - environmental modifications; specifically, the addition of portable play equipment [61] as well as the provision of staff training [34]. The modification to the required outdoor playtime schedules at centre-based childcare facilities has been a less common approach to childcare interventions. To our knowledge, only a few interventions have adopted a similar approach and the results remain ambiguous. Wolfenden and colleague’s [38] intervention divided the typical single 45-min bout of outdoor playtime afforded in Australian childcare centres into three shorter outdoor sessions of 15 min each; however, no results have been published to date. Similar to the SPACE intervention, Alhassan and colleagues [62] also implemented four 30-min outdoor play sessions in a small number of childcare centres in the United States. While these researchers found no significant effect of their intervention on Latino preschoolers’ MVPA, it is possible this was a consequence of the short implementation timeframe of only two days. The SPACE intervention’s success at improving preschoolers’ MVPA is noteworthy given the many health benefits associated with higher intensity activity, and the notion that most childcare interventions to date have been more effective at increasing lower intensity activity among this population [34]. In light of the significant (albeit short-term) changes in MVPA, TPA, and sedentary time among preschoolers in the SPACE intervention, additional research is needed to confirm the utility of a modified outdoor playtime schedule in childcare centres as a mechanism to support increased activity participation.

Researchers have previously identified outdoor playtime as a strong correlate of young children’s physical activity [63, 64]. In fact, Vanderloo and colleagues [27] noted that preschoolers were two times more active outdoors than indoors, averaging 31.68 min/h compared to 14.42 min/h outdoors versus indoors, respectively. With consideration of higher intensity activity, these same researchers reported that preschoolers’ MVPA levels were 10 times higher outdoors compared to indoors. Moreover, as noted previously, researchers have found that preschoolers display the most physical activity during the first 10 min outdoors [29, 65] and that activity is most intense during this time [30]. To capitalize on this peak in activity participation for this cohort, a modified outdoor playtime schedule was afforded to preschoolers in the SPACE intervention; specifically, four shorter outdoor sessions, compared to the typical two sessions (preschoolers’ total outdoor time did not change – 2 h per day). Given the intermittent nature of young children’s activity behaviours [66], offering more frequent, but shorter outdoor playtime sessions, appears to be a viable approach for promoting improved activity behaviours. Despite noted challenges with implementing increased outdoor playtime sessions within their current curriculum, our process evaluation, which captured attendance, adherence, dose delivered, content and feasibility, noted an adherence rate (over 70%) to these outdoor sessions in the SPACE intervention was high (Driediger MV, Vanderloo LM, Burke SM, Irwin JD, Gaston A, Timmons BW, et al. The feasibility and appropriateness of the supporting physical activity in the childcare environment (SPACE) intervention: a process evaluation. Health Educ Behav. Submitted). This suggests that the modified playtime schedule was feasible for childcare staff to follow.

In addition to significantly increasing physical activity levels, the SPACE intervention was also effective at decreasing preschoolers’ sedentary time. This finding is similar to Goldfield et al. [34] who also displayed significant decreases in sedentary time as a consequence of their childcare-based intervention. De Craemer and colleagues [33], who implemented the ToyBox-study, a kindergarten-based, family involved, intervention in over 800 Belgian preschoolers, found no significant decreases in sedentary time among their sample. However, a decrease in objectively and subjectively measured sedentary time was observed among preschoolers from the intervention group who were enrolled in high socioeconomic status kindergartens. A great deal of attention has recently been placed on sedentary behaviours and their associated negative health consequences; therefore, the effectiveness of the SPACE intervention to decrease this behaviour is noteworthy [10]. The replacement of sedentary time with MVPA, as observed in the present study, is a promising first step.

Although the SPACE intervention was successful at increasing activity behaviours and decreasing sedentary time while the program was running, these increases were not sustained long-term. Of note, the 6-month follow-up measures were conducted during the winter months in Canada where snow and extreme cold temperatures may limit outdoor time, which may negatively impact physical activity [67, 68]. The childcare centres assigned to the intervention condition kept the portable play equipment for use post-intervention and the childcare staff received a booster session at 4 months post-intervention to refresh their knowledge on the importance of physical activity for young children. As such, it seems possible that the outdoor playtime modification, more so than the provision of equipment and staff training, is the most promising for improving physical activity participation in childcare. However, this will require additional study to determine the independent effects of outdoor playtime modification.

While the present study has many strengths including its study design (i.e., clustered RCT), unique combination of evidence-informed components, and use of objective assessment tools (i.e., Actical™ accelerometers), it is not without limitations. We lost participants at follow-up (6- and 12-months) due to attrition, which occurred due to absences or withdrawals from childcare. While every effort was made to recruit participants who anticipated being in childcare for the next 12 months, the transient nature of childcare and the long study follow-up period at a very young age (with many of these children transitioning into elementary school) was problematic. Moreover, part time enrolment (e.g., half days) meant some participants were not included in the analysis due to inadequate accelerometer wear-time. Despite these limitations, this study provides insightful information about the potential of modifying outdoor playtime as a feasible approach to supporting preschoolers’ physical activity.

## Conclusion

The SPACE intervention was effective at improving rates of MVPA and TPA, while reducing sedentary time after the 8-week intervention; however, this effect was not sustained at 6- or 12-month follow-up. While the intervention was successful in the short-term, given the lack of long-term impact, it is possible that that modified outdoor playtime schedule, more so than the staff training and environmental modifications influenced changes in activity behaviours. Additional research is needed to determine whether a restructuring of outdoor play periods alone can produce an increase in preschoolers’ physical activity levels.

## Abbreviations

CONSORT: Consolidated Standards of Reporting Trials
ISRCTN: International Standard Randomised Controlled Trial Number
LPA: Light physical activity
MVPA: moderate-to-vigorous physical activity
RCT: Randomized controlled trial
REB: Research ethics board
SPACE: Supporting Physical Activity in the Childcare Environment
TPA: Total physical activity
